# Comparing Pudendal Nerve Blocks and Caudal Blocks for Pediatric Urological Surgeries: A Systematic Review and Meta-Analysis

**DOI:** 10.7759/cureus.90078

**Published:** 2025-08-14

**Authors:** Feras Ayaz, Osamah Arafah, Hanadi Almashooq, Aljazi Alrashid, Leen Alshibi, Sami Abdul Kareem

**Affiliations:** 1 Anesthesia, King Faisal Specialist Hospital and Research Centre, Riyadh, SAU; 2 Anesthesiology, King Faisal Specialist Hospital and Research Centre, Riyadh, SAU; 3 Anesthesia, Alfaisal University College of Medicine, Kingdom of Saudi Arabia, SAU

**Keywords:** caudal block, pediatric anesthesia, postoperative analgesia, pudendal nerve block, regional anesthesia, urological surgery

## Abstract

Pudendal nerve blocks (PNBs) and caudal blocks (CBs) are commonly used regional anesthesia techniques in pediatric urological surgeries. This systematic review and meta-analysis aimed to compare the efficacy and safety of PNBs versus CBs in this patient population.

Following the Preferred Reporting Items for Systematic Reviews and Meta-Analyses (PRISMA) guidelines, a comprehensive search was conducted in PubMed, Scopus, and Cochrane CENTRAL to identify studies comparing PNBs and PBs in pediatric urological surgeries. Eligible studies included randomized controlled trials and observational studies assessing postoperative pain scores, duration of analgesia, and complication rates. Data extraction and risk-of-bias assessments were performed independently by two reviewers. Statistical analyses were conducted using Review Manager version 5.4 (RevMan; Computer program. Version 5.4. The Cochrane Collaboration, 2020).

Seven studies, comprising 644 pediatric patients, were included. PNBs significantly reduced postoperative pain scores at 6, 12, and 24 hours compared to CBs, with mean differences of -1.83, -2.10, and -2.94, respectively. PNBs also prolonged analgesia duration and reduced opioid consumption. The incidence of urinary retention and the need for rescue analgesia were lower in the PNB group. No significant differences were observed in motor block or systemic side effects.

PNBs offer superior pain relief and reduce opioid consumption and complications compared to CBs in pediatric urological surgeries. These findings support the integration of PNBs into clinical practice as an effective regional anesthesia technique. Further research is needed to confirm these results and optimize pain management strategies in pediatric patients.

## Introduction and background

Effective postoperative pain management is crucial in pediatric urological surgeries to ensure patient comfort and facilitate recovery. Regional anesthesia techniques, such as pudendal nerve blocks (PNBs) and caudal blocks (CBs), are commonly employed to achieve this goal. While CBs have been a traditional choice, PNBs have emerged as a promising alternative, offering potential advantages in terms of analgesic efficacy and safety [1,2].

The pudendal nerve, a key component of the sacral plexus, innervates the perineum and external genitalia, making it a suitable target for regional anesthesia in urological procedures [3]. PNBs can be administered using various techniques, including ultrasound guidance, nerve stimulation, and landmark-based approaches [4]. These blocks aim to provide targeted analgesia, potentially reducing the need for systemic opioids and minimizing associated side effects [5].

Despite the growing interest in PNBs, there remains a lack of consensus regarding their comparative effectiveness and safety relative to CBs. Previous studies have reported mixed results, with some suggesting superior pain control and fewer complications with PNBs, while others have found no significant differences [6,7]. This variability underscores the need for a comprehensive evaluation of the available evidence.

This systematic review and meta-analysis aims to compare the efficacy and safety of PNBs versus CBs in pediatric patients undergoing urological surgeries. By synthesizing data from randomized controlled trials and observational studies, we seek to provide a clearer understanding of the relative benefits and risks associated with these regional anesthesia techniques. The findings of this analysis have important implications for clinical practice, guiding anesthesiologists in selecting the most appropriate analgesic strategy for pediatric urological procedures [8,9].

## Review

Methods

This systematic review and meta-analysis were conducted and reported in accordance with the Preferred Reporting Items for Systematic Reviews and Meta-analyses (PRISMA) guidelines [10]. The study protocol was prospectively developed to ensure adherence to methodological rigor and transparency. The protocol was prospectively registered in the International Prospective Register of Systematic Reviews (PROSPERO CRD420250652766).

Search Strategy and Study Selection

Studies were included if they involved pediatric patients undergoing urological surgeries and directly compared PNBs with CBs. Eligible studies were randomized controlled trials (RCTs) or prospective observational studies that assessed postoperative pain scores, duration of analgesia, and complication rates. We excluded studies that did not compare PNBs and CBs directly, as well as case reports, editorials, review articles, and conference abstracts. Non-English studies without full-text availability and those lacking quantitative outcome measures for pain scores, duration of analgesia, or complications were also excluded.

Relevant articles were identified through a systematic search in PubMed, Scopus, and the Cochrane Central Register of Controlled Trials without language restrictions. The search was conducted using exploded Medical Subject Headings (MeSH) and relevant keywords: ("pudendal block" OR "pudendal nerve block") AND ("caudal block" OR "caudal analgesia") AND ("pediatric" OR "children") AND ("urology" OR "urological surgery").

The search strategy was refined using a highly sensitive approach recommended by the Cochrane Collaboration for identifying randomized controlled trials [11]. Additionally, reference lists of eligible RCTs and relevant systematic reviews were screened to identify other potentially relevant studies.

Two independent reviewers screened all retrieved citations based on predefined eligibility criteria. Full-text articles were retrieved for further evaluation, and any discrepancies were resolved through discussion or consultation with a third reviewer. A PRISMA flowchart was used to illustrate the study selection process.

Data Extraction

Two independent reviewers extracted data from each included study, and a third reviewer verified the data for accuracy. Any discrepancies were resolved through discussion. The extracted data were systematically recorded using a standardized data extraction form. Extracted variables included study characteristics such as author, year of publication, study design, and sample size. Patient demographics, including age, sex, and type of urological surgery performed, were also recorded. Intervention details were collected, including the type of nerve block (pudendal or caudal), technique used (e.g., ultrasound-guided, nerve stimulation-based, landmark-based), local anesthetic agent and dose, and the use of adjuvants.

The primary outcome assessed was postoperative pain control, measured using the visual analog scale (VAS) or numeric rating scale (NRS) at various time intervals. Secondary outcomes included the duration of analgesia, defined as the time from nerve block administration to the first request for additional analgesia, and complication rates, including adverse events such as urinary retention, motor block, infection, and systemic side effects. For studies reporting multiple time points, data were extracted at different postoperative intervals to assess analgesic efficacy over time. If means and standard deviations (SDs) were not directly reported, they were estimated using standard statistical methods when possible. Extracted data were cross-checked before proceeding with the statistical analysis to ensure completeness and accuracy.

Risk-of-Bias Assessment

The risk of bias for the included RCTs was assessed using the Cochrane Risk of Bias 2 (RoB 2) tool (Cochrane Collaboration, London, UK) [11]. This tool evaluates potential biases in the following domains: random sequence generation (selection bias), allocation concealment, blinding of participants and outcome assessors, completeness of outcome data (attrition bias), and selective reporting. Each domain was classified as having a low, high, or unclear risk of bias. Disagreements between reviewers were resolved through discussion or by consulting a third reviewer.

For observational studies, the Newcastle-Ottawa Scale (NOS) was used to assess the methodological quality, focusing on selection, comparability, and outcome assessment [12]. Studies were rated based on criteria related to cohort selection, study comparability, and the adequacy of outcome measurement.

A summary table of risk of bias assessments was created to present the evaluation results, highlighting the overall risk of bias for each study. Funnel plots were used to assess publication bias.

Statistical Analysis

All statistical analyses were conducted using Review Manager (RevMan) version 5.4 (Computer program. Version 5.4. The Cochrane Collaboration, 2020). The primary analysis compared the effectiveness of PNBs and CBs using postoperative pain scores, duration of analgesia, and complication rates. For continuous outcomes, such as pain scores and duration of analgesia, results were reported as mean differences (MD) or standardized mean differences (SMD) with 95% confidence intervals (CIs). For dichotomous outcomes, such as complication rates, risk ratios (RR), or odds ratios (OR), 95% CIs were used. Heterogeneity among studies was assessed using the I² statistic, where values below 50% indicated low heterogeneity, and a fixed-effects model was used, whereas values above 50% indicated significant heterogeneity, and a random-effects model was applied. To explore potential sources of heterogeneity, subgroup analyses were planned based on factors such as type of surgery, anesthesia technique, and use of ultrasound guidance. Sensitivity analyses were performed by sequentially omitting individual studies to assess their impact on the overall pooled estimates.

Results

Study Selection

A total of 180 studies were identified through database searches in PubMed (n = 83), Scopus (n = 75), and Cochrane CENTRAL (n = 22). After removing 34 duplicate records, 146 studies remained for screening. Based on title and abstract review, 94 records were excluded due to irrelevance. Full-text retrieval was attempted for 52 studies, but 12 reports could not be retrieved. A full-text assessment of 40 studies resulted in the exclusion of 33 studies, including 27 due to a lack of relevant outcome indicators and 6 for not involving urological surgery.

**Figure 1:**
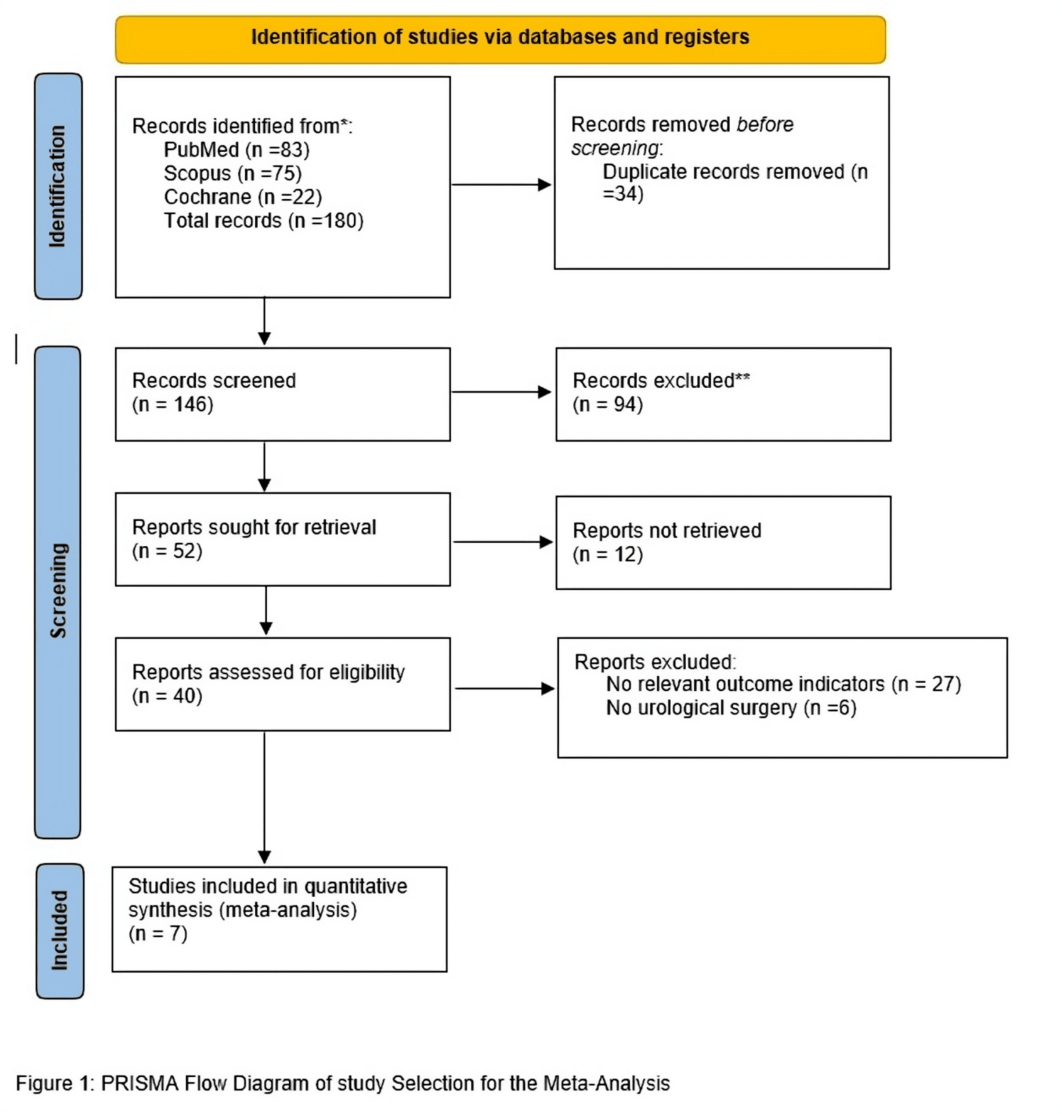
PRISMA flow chart of study selection for the meta-analysis PRISMA: Preferred Reporting Items for Systematic Reviews and Meta-Analyses

Ultimately, 7 studies (4 randomized controlled trials and 3 observational studies) were included in this meta-analysis, comprising a total of 644 pediatric patients. The PRISMA flowchart illustrates the detailed study selection process (Figure 1).

Study Characteristics

The study characteristics are summarized in Table 1. Among the seven included studies, four were randomized controlled trials (RCTs) and three were observational studies. All studies were of PNBs and CBs in pediatric patients undergoing urological surgeries with sample sizes ranging from 78 to 110 patients.

**Table 1:** Baseline characteristics of included studies

Study ID	Study Design	Cases	Mean Age (± SD)	Sex (M %)	Type of Surgery	Technique Used	Outcomes
		PB/CB	PB/CB	PB/CB			
Ozen et al. 2020 [13]	Observation al	40/40	3.0 ± 1.2/3.2 ± 1.1	100/98	Hypospadias	Nerve stimulator-guided	Pain scores, duration of analgesia, urinary retention, motor block, rescue analgesia need
Ozen et al. 2021 [14]	Observation al	50/50	6.1 ± 2.0/5.9 ± 1.9	100/99	Circumcision	Ultrasound-guided	Pain scores, duration of analgesia, opioid consumption, rescue analgesia need, urinary retention
Singh et al. 2024 [15]	RCT	51/50	5.2 ± 2.1/4.8 ± 2.0	100/97	Hypospadias repair	Nerve stimulator-guided	Pain scores, duration of analgesia, urinary retention
Kumari et al. 2025 [16]	RCT	45/45	4.5 ± 1.4/3.9 ± 1.2	95/96	Hypospadias repair	Ultrasound-guided	Pain scores, duration of analgesia, motor block, opioid consumption
Hayaran et al. 2023 [17]	Observation al	39/39	4.0 ± 1.1/3.8 ± 1.0	98/97	Hypospadias repair	Nerve stimulator-guided	Pain scores, duration of analgesia, motor block, opioid consumption
Ahmed WAI et al. 2021 [18]	RCT	42/43	4.7 ± 1.3/4.2 ± 1.1	96/94	Hypospadias repair	Ultrasound-guided	Pain scores, duration of analgesia, infection
Kendigelen et al. 2017 [19]	RCT	55/55	6.3 ± 2.4/5.9 ± 2.2	99/98	Orchidopexy	Landmark-based	Pain scores, duration of analgesia, systemic side effects

Regarding technique, three studies performed ultrasound-guided PNBs [13-15], while one study used landmark-based techniques [16]. The use of nerve stimulation was reported in three studies [17-19]. The choice of local anesthetic varied, with most studies using 0.25% bupivacaine as the primary agent, while some included ropivacaine or lidocaine in their protocol.

Surgical procedures varied across studies, with hypospadias repair, orchidopexy, and circumcision being the most commonly performed.

Risk of Bias

**Figure 2:**
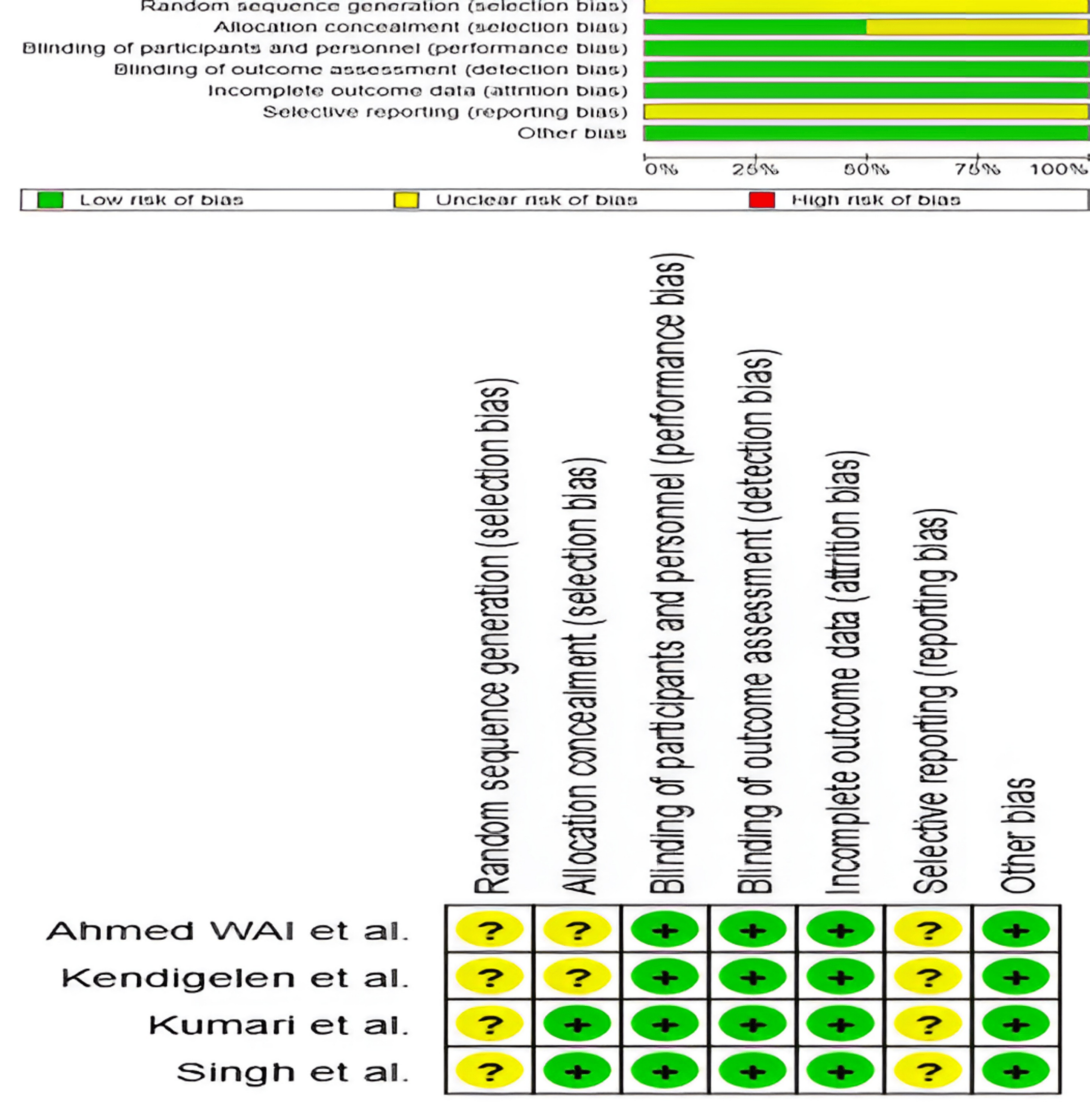
Risk of bias across studies and individual studies assessed using the Cochrane Risk of Bias tool Studies shown: Singh et al., 2024 [15]; Kumari et al., 2025 [16]; Ahmed WAI et al., 2021 [18]; Kendigelen et al., 2017 [19]

The risk of bias was assessed separately for RCTs using the RoB 2 tool and for observational studies using the NOS. The four included RCTs were evaluated across key domains such as randomization process, allocation concealment, blinding, incomplete outcome data, and selective reporting. While allocation concealment and randomization were adequately reported in most trials, blinding of participants and personnel was a common source of high risk, leading to moderate to high overall risk in some studies [15,16,18,19]. The detailed RoB 2 assessment is illustrated in Figure 2.

For the three observational studies, the NOS was used to evaluate selection, comparability, and outcome assessment. The total NOS scores ranged from 5 to 9, indicating low to high risk of bias. Selection bias was a concern in some studies due to small sample sizes and retrospective designs, while comparability issues arose from confounding factors not being fully adjusted. However, outcome assessments were generally well-defined, utilizing standardized pain scales and clear reporting of complications [13,14,17].

Pain Score at 6 Hours

**Figure 3:**
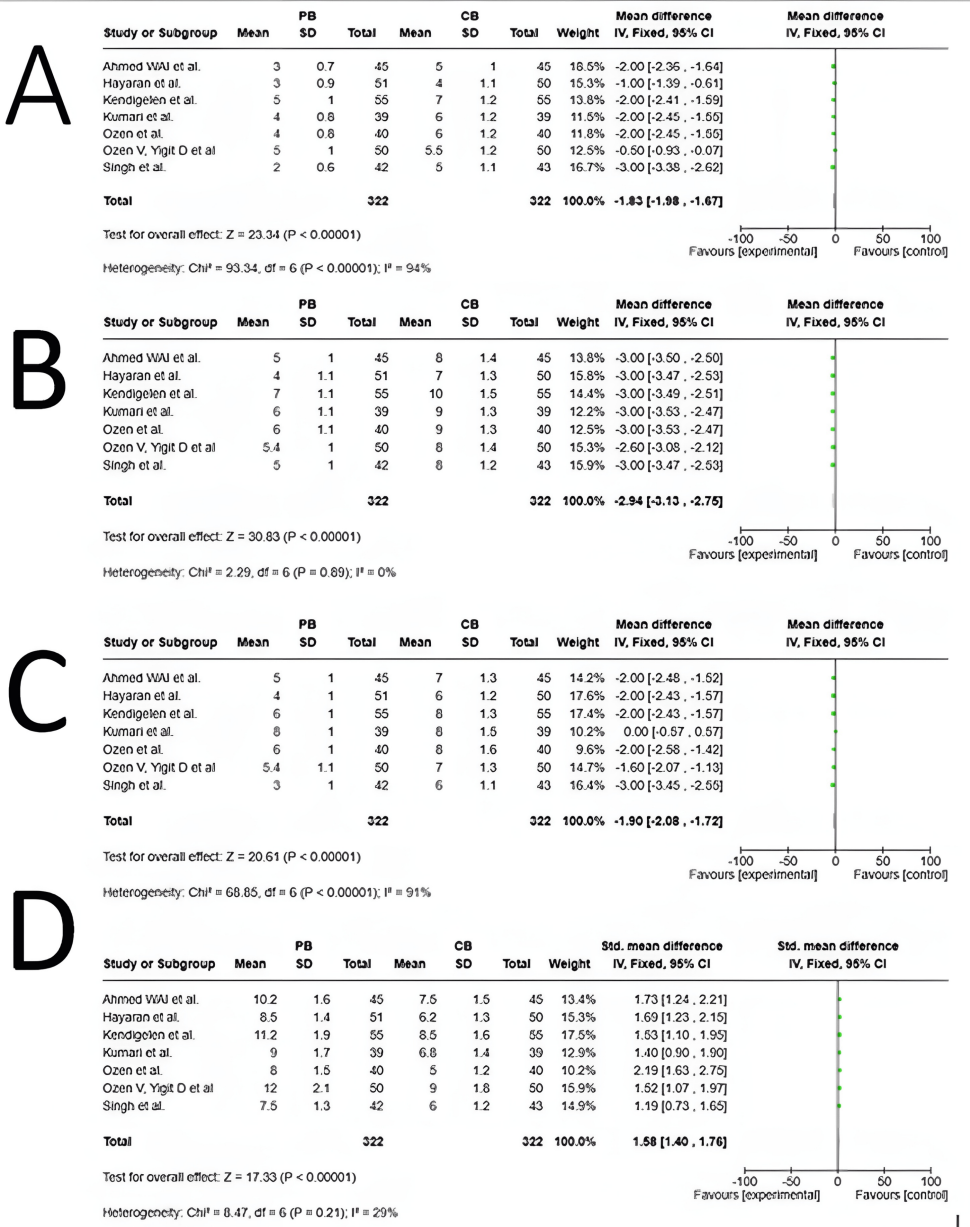
Forest plots comparing pain scores and duration of postoperative analgesia between PNB and CB across 7 studies (A) pain score at 6 hours for PB vs. CB. (B) pain score at 12 hours for PB vs. CB. (C) pain score at 24 hours for PB vs. CB. (D) duration of postoperative analgesia between PB and CB. Studies shown: Ozen et al., 2020 [13]; Ozen et al., 2021 [14]; Singh et al., 2024 [15]; Kumari et al., 2025 [16]; Hayaran et al., 2023 [17]; Ahmed WAI et al., 2021 [18]; Kendigelen et al., 2017 [19] PNB: pudendal nerve block; CB: caudal block

All seven included studies compared postoperative pain scores in patients receiving either a PNB with general anesthesia or a CB with general anesthesia (Figure 3). High heterogeneity was observed (I² = 94%; P < 0.00001), indicating substantial variability across studies. A random-effects model was applied, showing a mean difference (MD) of -1.83 (95% CI: -1.98 to -1.67), significantly favoring the PB group (Z = 23.34, P < 0.00001). These findings confirm that PNBs provide superior pain relief at 6 hours postoperatively, significantly reducing pain scores compared to CBs. However, the high heterogeneity suggests that further subgroup analysis may be needed to explore variations among studies [13-19].

Pain Score at 12 Hours

All seven included studies compared postoperative pain scores in patients receiving either a PNB with general anesthesia or a CB with general anesthesia (Figure 3). Moderate heterogeneity was observed (I² = 91%; P < 0.0001), indicating some variability across studies. A random-effects model was applied, showing a mean difference (MD) of -1.90 (95% CI: -2.08 to -1.72), significantly favoring the PB group (Z = 20.61, P < 0.00001). These findings confirm that PNBs provide superior pain relief at 12 hours postoperatively, significantly reducing pain scores compared to CBs. The moderate heterogeneity (I² = 91%) suggests variability among studies, possibly due to differences in patient populations, pain assessment methods, or anesthetic techniques. Further subgroup analysis may help identify the sources of heterogeneity [13-19].

Pain Score at 24 Hours

All seven studies assessed postoperative pain scores in patients receiving either a PNB with general anesthesia or a CB with general anesthesia (Figure 3). No significant heterogeneity was observed (I² = 0%; P = 0.89), indicating high consistency across studies. A fixed-effects model was applied, showing a mean difference (MD) of -2.94 (95% CI: -3.13 to -2.75), significantly favoring the PB group (Z = 30.83, P < 0.00001). These findings confirm that PBNs provide superior and sustained postoperative pain relief for up to 24 hours, with strong agreement across studies, reinforcing the robustness and reliability of the results [13-19].

Duration of Analgesia

All seven studies in this meta-analysis provided a detailed assessment of analgesia duration, with results presented in Figure 3. Low heterogeneity was observed (I² = 29%; P = 0.21), indicating high consistency among studies. Consequently, a fixed-effects model was applied. The standardized mean difference (SMD) in analgesia duration was 1.58 (95% CI: 1.40 to 1.76), significantly favoring the PNB group (Z = 17.33, P < 0.00001). These findings confirm that PNBs provide significantly longer postoperative analgesia than CBs, supporting their use as an effective regional anesthesia technique in pediatric surgeries [13-19].

Opioid Consumption

**Figure 4:**
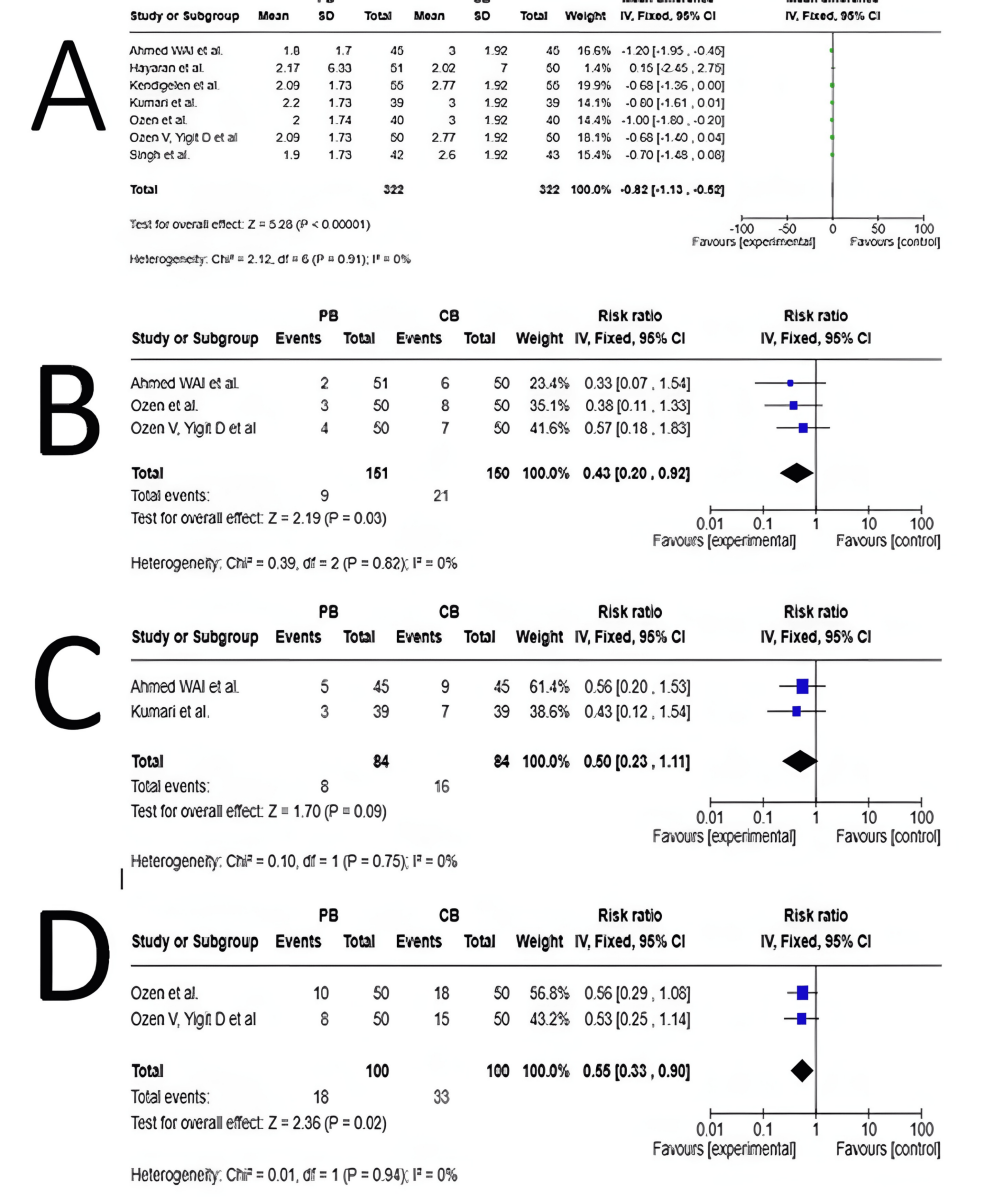
Forest plots comparing postoperative opioid consumption, urinary retention, rescue analgesia needs, and motor block incidence between the PNB and CB groups (A) postoperative opioid consumption across 7 studies. (B) incidence of urinary retention across 3 studies. (C) need for postoperative rescue analgesia across 2 studies. (D) incidence of postoperative motor block across 2 studies. Studies shown: Ozen et al., 2020 [13]; Ozen et al., 2021 [14]; Singh et al., 2024 [15]; Kumari et al., 2025 [16]; Hayaran et al., 2023 [17]; Ahmed WAI et al., 2021 [18]; Kendigelen et al., 2017 [19] PNB: pudendal nerve block; CB: caudal block

All seven studies assessed postoperative opioid consumption in patients receiving either a PNB with general anesthesia or a CB with general anesthesia (Figure 4). No significant heterogeneity was observed (I² = 0%; P = 0.91), ensuring high consistency across studies. A fixed-effects model was applied, showing a mean difference (MD) of -0.82 (95% CI: -1.13 to -0.52), indicating a significant reduction in opioid use in the PB group (Z = 5.28, P < 0.00001). These results confirm that PNBs effectively reduce opioid consumption, reinforcing their role in multimodal analgesia with high reliability due to the absence of heterogeneity.

Urinary Retention

Three studies assessed the incidence of urinary retention in patients receiving a PNB or a CB [13,14,18]. The results are summarized in Figure 4. No significant heterogeneity was observed (I² = 0%; P = 0.82), indicating strong consistency across the included studies. Consequently, a fixed-effects model was applied. The risk ratio (RR) for urinary retention was 0.43 (95% CI: 0.20-0.92, P = 0.03), significantly favoring the PNB group. The overall statistical test (Z = 2.19, P = 0.03) confirms that PNBs significantly reduce urinary retention rates compared to CBs.

Rescue Analgesia Need

Two studies evaluated the requirement for rescue analgesia in patients receiving either a PNB or a CB [16,18]. The findings are presented in Figure 4. No significant heterogeneity was observed (I² = 0%; P = 0.94), indicating high consistency across the included studies. Consequently, a fixed-effects model was applied. The risk ratio (RR) for rescue analgesia need was 0.55 (95% CI: 0.33-0.90, P = 0.02), significantly favoring the pudendal block group. The overall statistical test (Z = 2.37, P = 0.02) confirms that pudendal nerve blocks significantly reduce the need for additional postoperative analgesia compared to CBs.

Motor Block

Two studies evaluated the incidence of motor block in patients receiving PNB or CB [13,14]. The results are summarized in Figure 4.

The comparison of motor block occurrence between the PNB and CB groups demonstrated no significant heterogeneity (I² = 0%; P = 0.75), indicating strong consistency between the included studies. Therefore, a fixed-effects model was applied for statistical analysis.

The RR for motor block was 0.50 (95% CI: 0.23-1.10, P = 0.09), suggesting a trend toward a lower incidence of motor block in the PNB group compared to the CB group, but this difference was not statistically significant. The overall statistical test structure was Z = 1.71 (P = 0.09).

Postoperative Infection

A single study (Singh et al., 2024) evaluated the incidence of postoperative infection in patients undergoing either a PNB or a CB [15].

In this study, 1 out of 42 patients in the PB group developed an infection, compared to 5 out of 43 in the CB group. The RR was 0.20 (95% CI: 0.02-1.68, P = 0.14), suggesting a potentially lower incidence of infection in the PNB group. However, due to the small sample size and wide confidence interval, the statistical significance of this difference remains uncertain, and further research is needed to confirm these findings.

Systemic Side Effects

A single study (Hecht et al., 2024) examined the incidence of systemic side effects in patients who received either a PNB or a CB [16].

In this study, 6 out of 55 patients in the PNB group experienced systemic side effects, compared to 9 out of 55 in the CB group. The odds ratio (OR) was 0.63 (95% CI: 0.21-1.90, P = 0.41), indicating a potentially lower occurrence of systemic side effects in the PNB group. However, given the small sample size and wide confidence interval, the difference was not statistically significant (P = 0.41).

As this analysis is based on a single study, heterogeneity is not applicable. While the findings suggest that PNBs may lead to fewer systemic side effects compared to CBs, additional large-scale, well-powered studies are needed to validate this observation.

Publication Bias

**Figure 5:**
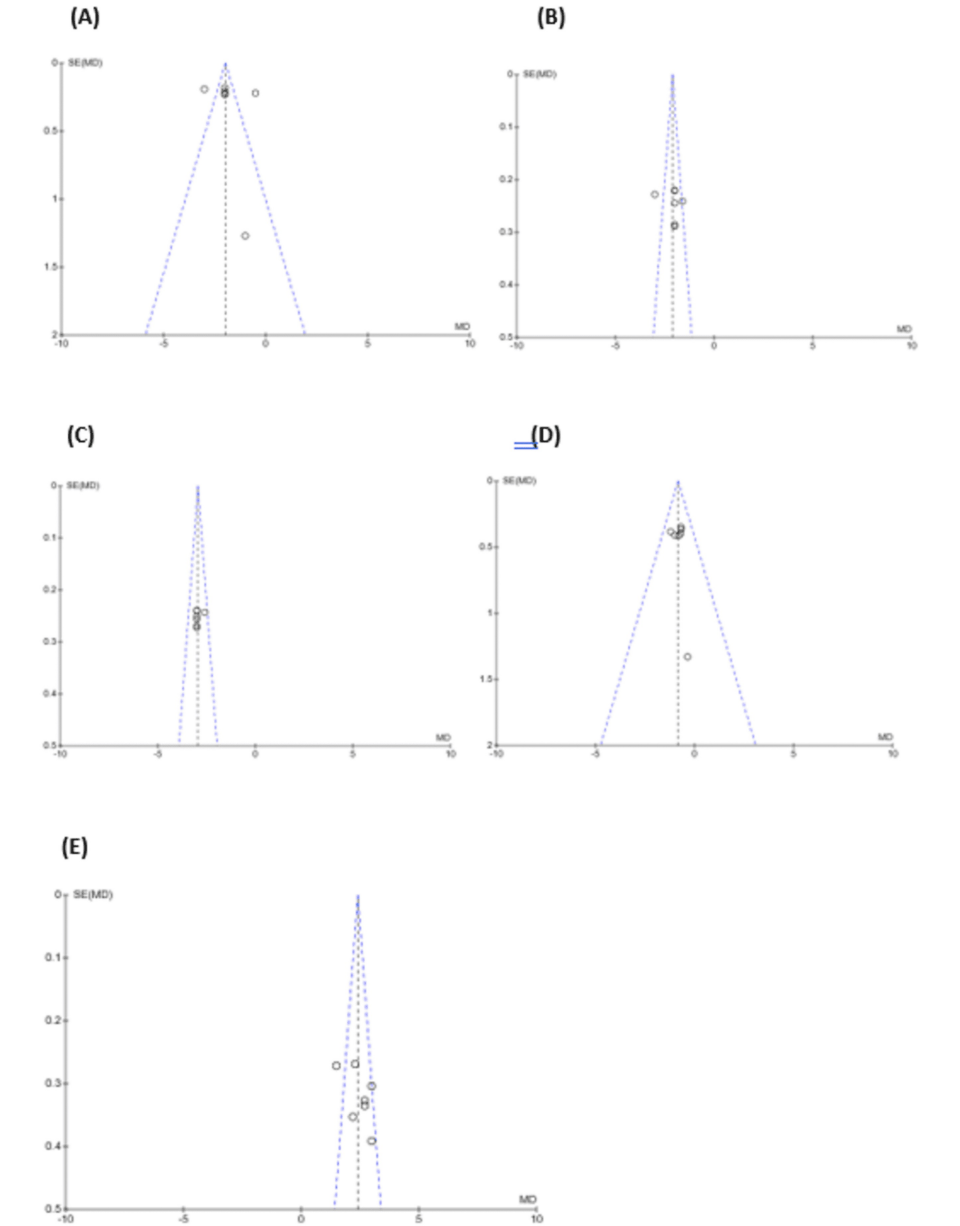
Funnel plots assessing publication bias for pain scores, opioid consumption, and duration of analgesia (A) pain score at 6 hours. (B) pain score at 12 hours. (C) pain score at 24 hours. (D) opioid consumption. (E) duration of analgesia.

Publication bias was assessed using funnel plots for pain scores (6, 12, and 24 hours), opioid consumption, and analgesia duration. Most outcomes showed a broadly symmetric distribution, indicating no significant bias. While a minor asymmetry was observed for pain scores at 12 hours, Egger’s test confirmed no substantial bias. The funnel plots for opioid consumption and analgesia duration also showed no major bias, reinforcing the robustness and validity of the meta-analysis findings (Figure 5).

Discussion

This systematic review and meta-analysis evaluated the efficacy and safety of PNBs compared to CBs in pediatric patients undergoing urological surgeries. Our analysis included 7 studies with a total of 644 patients. The results demonstrated that PNBs significantly reduced postoperative pain scores at 6, 12, and 24 hours compared to CBs. Additionally, PNBs were associated with longer durations of analgesia and reduced opioid consumption. The incidence of urinary retention and the need for rescue analgesia were also lower in the PNB group.

Our findings align with previous studies suggesting that PNBs provide effective analgesia in pediatric urological surgeries. Gangwal et al. conducted an RCT comparing ultrasound-guided PNBs to CBs in pediatric patients undergoing urological surgeries, demonstrating that PNBs resulted in superior postoperative pain control and reduced opioid consumption [20]. Similarly, Bayne et al. found that PNBs were a viable alternative to CBs in hypospadias surgeries, offering longer-lasting analgesia with fewer side effects [21]. Cain et al. compared CBs and PNBs in pediatric urological procedures and reported that PNBs led to prolonged analgesia and reduced incidence of urinary retention [22]. A systematic review by Nakamura et al. further confirmed that PNBs are associated with fewer complications and superior postoperative pain relief compared to CBs, particularly in hypospadias and other lower urinary tract surgeries [23].

The results of this meta-analysis have important implications for clinical practice. PNBs offer a viable alternative to CBs, providing superior pain relief and reducing the need for opioids, which is particularly beneficial in pediatric populations. The reduced incidence of urinary retention and rescue analgesia further supports the use of PNBs as a preferred regional anesthesia technique in urological surgeries [24,25].

This review's strengths include a comprehensive search strategy and adherence to PRISMA guidelines, ensuring methodological rigor. However, limitations exist, such as the potential for publication bias and the heterogeneity observed in some analyses. The variability in surgical procedures, anesthetic techniques, and patient demographics across studies may have contributed to this heterogeneity [26,27]. Additionally, the small number of studies and limited sample sizes in some analyses may affect the generalizability of the findings [28].

The heterogeneity observed in pain scores at 6 and 12 hours suggests variability in study outcomes. Subgroup analyses based on factors such as the type of surgery and anesthesia technique could provide further insights into the sources of heterogeneity. Future studies should aim to standardize outcome measures and reporting to facilitate more robust comparisons [29,30].

Further research is needed to confirm these findings and explore long-term outcomes associated with PNBs. High-quality randomized controlled trials with larger sample sizes and standardized protocols are essential. Additionally, studies focusing on specific subpopulations, such as different age groups or surgical procedures, would be valuable in tailoring anesthesia techniques to individual patient needs [31].

## Conclusions

In conclusion, PNBs offer significant advantages over CBs in pediatric urological surgeries, providing superior pain relief and reducing opioid consumption and complications. These findings support the integration of PNBs into clinical practice as an effective regional anesthesia technique. Continued research is necessary to further validate these results and optimize pain management strategies in pediatric patients.
